# Design of Low-Cost Vehicle Roll Angle Estimator Based on Kalman Filters and an IoT Architecture

**DOI:** 10.3390/s18061800

**Published:** 2018-06-03

**Authors:** Javier Garcia Guzman, Lisardo Prieto Gonzalez, Jonatan Pajares Redondo, Susana Sanz Sanchez, Beatriz L. Boada

**Affiliations:** 1 Computer Science Department, Institute for Automotive Vehicle Safety (ISVA), Universidad Carlos III de Madrid, Avda. de la Universidad 30, 28911 Leganés, Madrid, Spain; lpgonzal@inf.uc3m.es; 2 Mechanical Engineering Department, Institute for Automotive Vehicle Safety (ISVA), Universidad Carlos III de Madrid, Avda. de la Universidad 30, 28911 Leganés, Madrid, Spain; jopajare@ing.uc3m.es (J.P.R.); ssanz@ing.uc3m.es (S.S.S.); bboada@ing.uc3m.es (B.L.B.)

**Keywords:** real-time estimation, IoT, MEMS, low cost devices, Kalman Filter, vehicle dynamics, roll angle

## Abstract

In recent years, there have been many advances in vehicle technologies based on the efficient use of real-time data provided by embedded sensors. Some of these technologies can help you avoid or reduce the severity of a crash such as the Roll Stability Control (RSC) systems for commercial vehicles. In RSC, several critical variables to consider such as sideslip or roll angle can only be directly measured using expensive equipment. These kind of devices would increase the price of commercial vehicles. Nevertheless, sideslip or roll angle or values can be estimated using MEMS sensors in combination with data fusion algorithms. The objectives stated for this research work consist of integrating roll angle estimators based on Linear and Unscented Kalman filters to evaluate the precision of the results obtained and determining the fulfillment of the hard real-time processing constraints to embed this kind of estimators in IoT architectures based on low-cost equipment able to be deployed in commercial vehicles. An experimental testbed composed of a van with two sets of low-cost kits was set up, the first one including a Raspberry Pi 3 Model B, and the other having an Intel Edison System on Chip. This experimental environment was tested under different conditions for comparison. The results obtained from low-cost experimental kits, based on IoT architectures and including estimators based on Kalman filters, provide accurate roll angle estimation. Also, these results show that the processing time to get the data and execute the estimations based on Kalman Filters fulfill hard real time constraints.

## 1. Introduction

In recent years, there have been many advances in vehicle technologies based on the efficient use of real-time data provided by embedded sensors. Some of these technologies can help you avoid or reduce the severity of a crash such as the Roll Stability Control (RSC) systems for commercial vehicles [1,2,3]. These systems contribute to monitor and improve the vehicle stability, comfort and handling. This control functionality requires knowing relevant information of the vehicle dynamics, such as angular rates, angular positions, lateral and longitudinal acceleration [4,5,6],

Several related measures (lateral and longitudinal acceleration, yaw rate or roll rate) can be measured using low cost sensors, but others (i.e., sideslip or roll angle) need to be obtained using very expensive equipment such as Global Positioning System (GPS) dual antenna; they cannot be measured using other sensors [7]. These kind of GPS devices would increase the price of commercial vehicles.

Nevertheless, sideslip or roll angles can be estimated using data provided by low-cost devices to solve the price problem in an efficient way. Several studies estimate sideslip applying data fusion techniques using low-cost GPS, inertial navigation systems, yaw rate and lateral acceleration [4,5].

In a similar way, roll angle can be estimated by the fusion of wheel angular speed, yaw rate, steering angle as proposed in [8] or fusing yaw rate, roll rate, lateral and longitudinal acceleration as in discussed in [7]. In most cases, the data required for this kind of proposals is obtained using a six-dimensional IMU [9]. The data fusion algorithms considered to estimate roll angle are based on different techniques implemented separately or jointly: Bayesian Filters [8], Kalman Filters [3,9,10], robust observers [6,11,12] and neural networks [3,7].

The Internet of Things (IoT) refers to the interconnection of uniquely-identifiable embedded devices within the Internet infrastructure [13]. IoT technologies are able to contribute to the effective implementation of real-time RSC systems embedded in commercial vehicles providing added value services, integrating a large amount of sensors, actuators, and communication devices (mobile devices, GPS devices, and embedded computers [14,15,16]) into vehicles.

The main elements to consider in a IoT architecture to implement embedded RSC systems for vehicles are [17]:

1. The first architectural component of IoT is the perception layer. It collects data using sensors, which are the most important drivers of the Internet of Things [18].
2. The next architectural component that we shall discuss is communication. The most common communication technologies for vehicular communications are Bluetooth, Zigbee, and WiFi [19].
3. The last architectural component integrates two kinds of software elements: middleware and applications. Middleware enhances interoperability of smart things and makes it easy to offer different kinds of services [15,16,20,21,22,23]. The applications include all the services notifying drivers roll risks situations and sending the appropriate orders to the vehicle active safety systems.

Nevertheless, it is necessary to consider specific requirements that IoT solution must satisfy to provide actual operational systems for RSC. These requirements include: (a) the data from the sensors in a high frequency sampling bases, at least at 50 Hz [14]; (b) in order to keep reasonable costs of commercial vehicles, it is necessary to obtain this information from low-cost sensors; (c) the data that cannot be provided directly by low-cost sensors, such as roll angle or side slip, must be estimated in a hard real time basis; (d) reduce the energy consumption to monitor the vehicle dynamics while the vehicle is running; and (e) the middleware must integrate in a synchronized, fault tolerant and reliable way the information coming from sensors and estimators.

Intel Edison and Raspberry Pi are examples of low-cost devices used to deploy IoT architectures in automotive research area [24,25]. These kinds of devices are widely used to implement IoT architectures due to their low price, flexibility and the knowledge available in the Internet to solve implementation problems [26,27,28,29]. These devices can be enriched with MEMS sensors (i.e., accelerometers and gyroscopes) to obtain the raw data required to properly estimate roll angle in real time [14,18].

As stated before, there are several types of roll angle estimators. In this research work, the estimators considered are based on Kalman techniques. A Linear Kalman filter can be used in applications that require real-time data fusion approaches because it is simple to implement, can be tailored to different sensor configurations, and provides accurate estimations in environments where the sensors provide raw data with noise [3]. Several practical applications of Kalman filters in research works in the automotive area are related to the estimation of vehicle roll angle [3,30,31], localization [32,33] and sideslip [10]. The Kalman filter is not able to handle curves as input. The Unscented Kalman filter introduces a workaround that transforms the curve to a bunch of points before executing the Kalman filter [34]. The difference between Linear and Unscented Kalman Filters is relatively small, but in the cases when the cumulative effect of small errors can lead to relevant errors in the estimations provided (such as vehicle roll angle estimation), the use of an Unscented Kalman Filter can be appropriate [35].

When embedding roll angle estimators in commercial vehicles implementing IoT principles, it is essential to take into account that these software components must fulfill strong, real time processing restrictions. Previous research works [14] suggest that effective implementations of neural networks implemented in high-performance programming languages such as C++ are able to fulfill hard real-time restrictions. Even more so, the performance levels achieved indicate the possibility to embed, in the low-cost experimental kits, more complex estimators using a sensor fusion approach to obtain roll angle estimations closer to the actual values based on Kalman filters or combining neural networks and Kalman filters.

According to previous discussion, the objectives stated for this research work consist of integrating roll angle estimators based on Linear and Unscented Kalman filters to evaluate the precision of the results obtained and determining the fulfillment of the hard real-time processing constraints to embed this kind of estimators in IoT architectures based on low-cost equipment able to be deployed in commercial vehicles and enrich the current roll stability control systems deployed in actual vehicles. In this sense, the novelty of this research is related to the evaluation of roll angle estimation using Kalman Filters embedded in an IoT architecture using low-cost sensors and devices in real time conditions. Raspberry Pi and Intel Edison low-cost systems were considered. The performance and accuracy of the estimations obtained in these two low-cost kits are compared. Even more, practical considerations and lessons learnt implementing these kinds of architectures are provided.

The sections in this article are organized as follows. Section 2 presents the testbed designed for this research work, the hypothesis definition, the experiments specification including data gathering and analysis approach. This section also includes a discussion of the threats to validity associated to the experimental approach stated. Section 3 presents the results regarding the precision and performance of the estimators provided. Finally, Section 4, the discussion and conclusion of the results and the method are exposed.

## 2. Methodology

This section describes the experimental approach adopted to achieve the goals stated for this research work. Section 2.1 describes the IoT testbed for roll angle estimation based on the application of Kalman Filter. Section 2.2 enumerates the hypothesis to evaluate and the experiments defined for this purpose. Section 2.3 introduces the data gathered during the experiments execution and the data analysis methods proposed to analyze the results obtained. Finally, Section 4 summarizes the threats to validity related to this research work.

### 2.1. IoT Testbed for Roll Angle Estimation Based in Kalman Filter

The testbed designed for this research work was designed to be installed in any vehicle, but in the case of this research work, it was deployed in a Mercedes Sprinter van. This vehicle was considered because it is necessary to evaluate the accuracy of the estimations provided by the Kalman filters with the results presented in [3].

**Figure 1:**
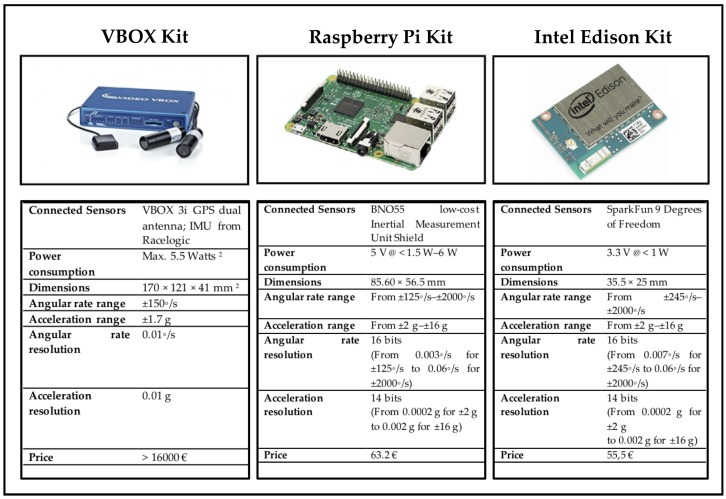
Technical specifications of hardware elements included in this study.

The considered testbed was composed of three experimental kits:

1. A ground truth kit using a VBOX 3i GPS dual antenna data logger with an IMU (Inertial Measurement Unit) from Racelogic. This sensors provide the reference measurements (roll angle roll and yaw, longitudinal and lateral acceleration).
2. A first low-cost experimental kit based on an Intel Edison chipset having connected a SparkFun “9 Degrees of Freedom” module.
3. A second low-cost kit using a Raspberry Pi 3 Model B with a Inertial Measurement Unit Shield. The technical specifications of this hardware elements are shown in Figure 1.

**Figure 2:**
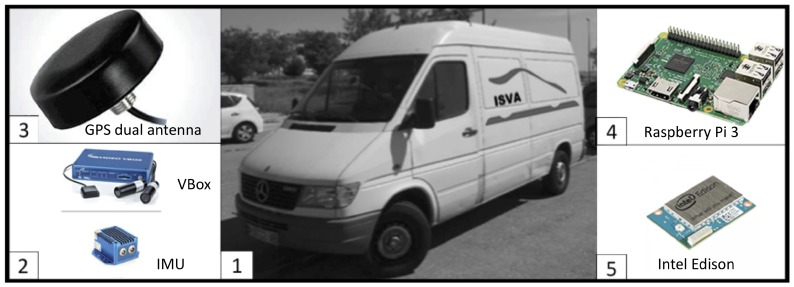
Experimental testbed installed in Mercedes Sprinter van (1) with the ground truth kit (2, 3), Raspberry Pi and Intel Edison kits (4, 5).

The testbed kit was deployed in a Mercedes Sprinter van. This deployment is shown in Figure 2.

**Figure 3:**
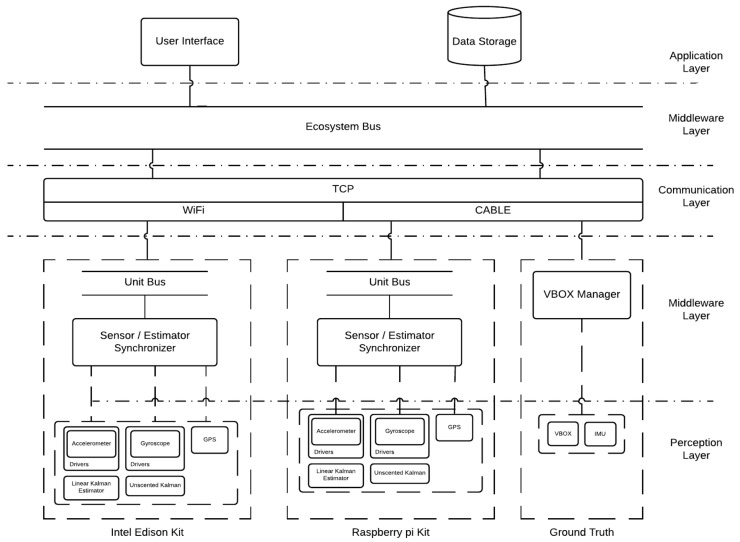
Testbed implemented IoT Architecture.

The considered testbed can be described using the levels of a typical IoT architecture [17]: application, middleware, communication and perception layers. These layers are presented in the following sections and summarized in Figure 3. The software components stated for each level are developed in C++ to optimize the performance as stated in [14].

#### 2.1.1. Application Layer

The application layer of this experimental kit is composed of a user interface that enables the experiments’ launch and finalization. This application uses the middleware functionality to send the requests to the kits integrated to execute the experiments with a full synchronization. Even more, it is in charge of storing the data sent from the ground truth and low-cost experimental kits in files in the CSV format.

#### 2.1.2. Middleware Layer

The software components considered in this layer provide the functionality required to execute the experiment and obtain the resulting data in a synchronized way. It is essential to obtain the raw data required to analyze the performance and accuracy regarding the roll-angle estimations using different types of Kalman filters.

**Figure 4:**
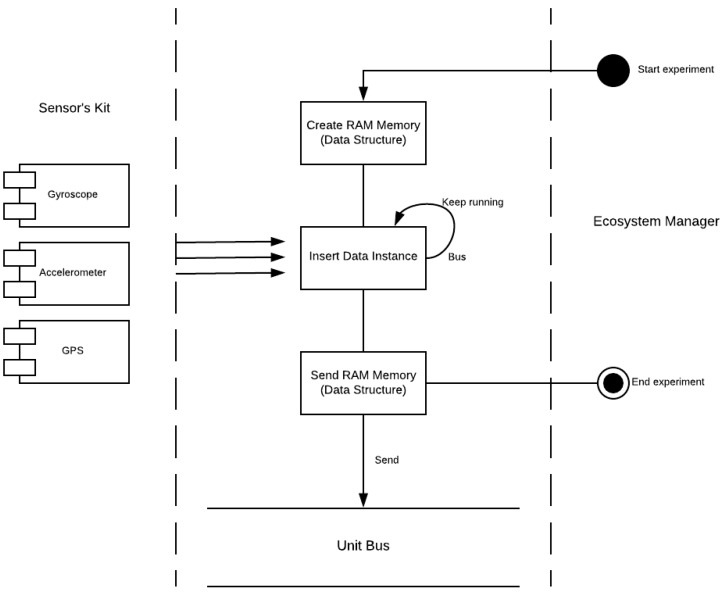
Sensors/Estimators Synchronizer functional diagram.

The middleware components are organized in different devices:

1. There is an Ecosystem Bus in charge of coordinating the experiments among all the experimental kits connected to the testbed. This Ecosystem Manager is deployed in a small computer able to be located in the experimental vehicle. This component provides the functionalities to connect and disconnect the experimental kits. Even more, it is in charge of sending requests to the low-cost kits to: (a) start an experiment; (b) continue running an experiment; (c) stop an experiment; and (d) shutdown an experimental kit. Finally, the ecosystem manager sends the data to the application layer in order to proceed to its storage.
2. The low cost experimental kits have their own middleware layer composed of two components:
  (a) The Unit Bus is in charge of coordinating each experimental kits with the Ecosystem Manager that is in charge of coordinating the whole testbed. The functionalities provided by the Unit Bus are: (a) publish the experimental kit in the testbed; (b) receive the requests from the Ecosystem Bus; (c) send the data obtained by the kit sensors to the Ecosystem Bus; and (d) send to the sensors synchronizer the experiments start and stop signals for gathering appropriately the data from the sensors and roll angle estimators.
  (b) The Sensors/Estimators Synchronizer obtains the data from the sensors and estimators with the required synchronization as it is shown in Figure 4. This component sends to the Unit Bus the data structure with the information gathered during the experiment when it receives the stop signal.
3. The middleware considered for the ground truth kit (VBOX based) consists of a software component (named VBOX Manager) that provides the functionality to manage the start/stop signals received from the Ecosystem Manager. This component also sends the data gathered from during the experiment execution. Due to the restrictions introduced by VBOX and Racelogic IMU manufacturers, the middleware component for the ground truth experimental kit is implemented in C#.

#### 2.1.3. Communication Layer

The communications layer has the responsibility to ensure homogeneous and synchronous communication among the kits included in the experimental testbed when the experiments are executed. This layer is implemented as follows:

1. Ground truth kit communications. The connection of the sensors and the laptop where the ground truth middleware is installed are connected using a cable due to the communication interfaces provided by the Racelogic IMU and VBOX Dual Antenna. The VBOX Manager and the Ecosystem manager are deployed in the same laptop.
2. Low-cost experimental kit communications. The low-cost experimental kits (based on Intel Edison and Raspberry Pi 3) are connected to the Ecosystem Manager using a WiFi connection through a wireless (802.11 g) access point. The connection between the sensors and the Sensors/Estimators Managers is implemented using the GPIO ports provided by the Intel Edison and Raspberry Pi development boards.

At logic level, the communications between the low-cost experimental kits and the laptop where the Ecosystem Manager is deployed are managed using TCP sockets.

#### 2.1.4. Perception Layer

This level is composed of the sensors and estimators connected to each experimental kit in the testbed:

1. The sensors considered in each experimental kit are implemented in a different way. As mentioned before the ground truth kit uses a Racelogic IMU and VBOX Dual Antenna. The drivers are provided by the manufacturers and used by the middleware element implemented in the VBOX Manager component. In the case of the low-cost experimental kits, each sensor (accelerometer and gyroscope) is managed through a driver implemented in C++ and used by the component that is deployed in the Intel Edison and Raspberry Pi development boards. These drivers gather the information from the sensors hardware using 50 Hz sampling rate.
2. The Roll Angle Estimator implements Linear and Unscented Kalman Filters to estimate roll angle in real time using a two Degree of Freedom (DoF) which represents the vehicle roll motion. A description of this model is presented in [35] and summarized in Section 2.1.5. As observation measurements required for both Kalman filters, lateral acceleration, roll rate and time are considered. Section 2.1.6 provides a more detailed description of this software component.
3. Finally, the NTP Client to assure the appropriate synchronization of the data gathered in the experimental kits included in the testbed, registers the actual date-time obtained from the GPS sensor in the hardware controller.

#### 2.1.5. Vehicle Model

For a better understanding of this section and Section 2.1.6, Table 1 shows all the variables for the model and estimators presented in this work.

**Figure 5:**
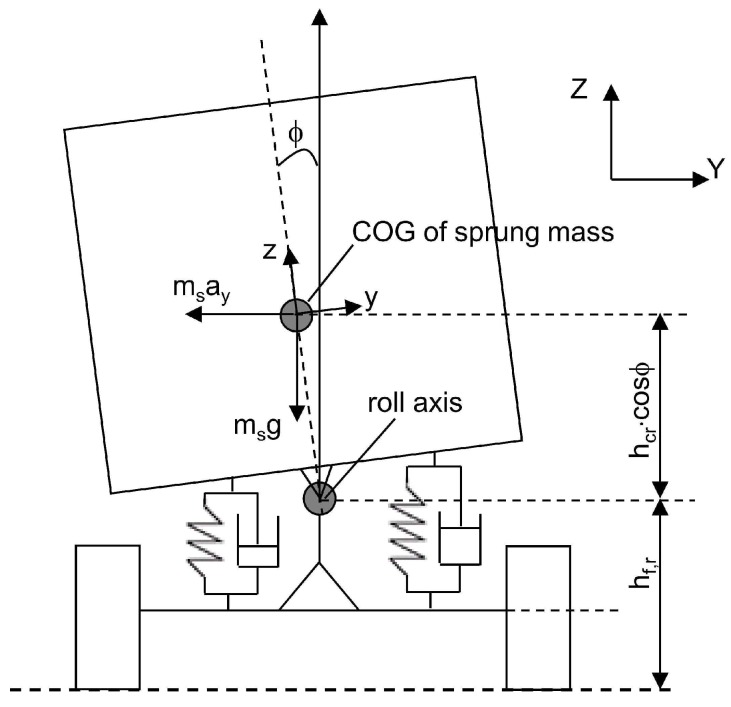
Vehicle roll model.

The model used in this research is presented in [35]. This model describes the roll vehicle motion in a discrete-time system (see Figure 5)

(1) $$\begin{array}{c} x_{k+1}=A_{d}x_{k}+B_{d}a_{ym,k}\\ y_{k}=Cx_{k} \end{array}$$

where:

(2) $$x_{k}={\left[\phi_{k}\;{\dot{\phi }}_{k}\right]}^{T}$$

(3) $$A_{d}=\left[\begin{array}{cc} 1+T_{s} & 0\\ -\frac{K_{r}\cdot T_{s}}{I_{xx}} & 1+\frac{C_{r}\cdot T_{s}}{I_{xx}} \end{array}\right]$$

(4) $$B_{d}=\left[\begin{array}{c} 0\\ \frac{m_{s}\cdot h_{cr\cdot T_{s}}}{I_{xx}} \end{array}\right]$$

(5) $$C=\left[\begin{array}{cc} 0 & 1 \end{array}\right]$$

The vehicle model constant values are presented in Table 2.

#### 2.1.6. Roll Angle Estimators based on Kalman Filters

Two different roll angle estimators based on Kalman filters were developed to achieve the research work defined:

1. First, a linear Kalman estimator was developed. This estimator has as inputs the actual roll rate, time and lateral acceleration. As result, the software component provides an estimated value of the current roll angle. The formulas implemented for the calculations are presented below [10]:
  (a) Prediction of state:
    (6) $${\bar{x}}_{k|k-1}=A_{d}{\bar{x}}_{k-1|k-1}$$
  (b) Prediction of error covariance:
    (7) $$P_{k|k-1}=A_{d}P_{k-1|k-1}A_{d}^{T}+Q$$
  (c) Kalman gain:
    (8) $$K_{k}=P_{k|k-1}H^{T}{\left[HP_{k|k-1}H^{T}+R\right]}^{-1}$$
  (d) State estimation:
    (9) $${\bar{x}}_{k|k}={\bar{x}}_{k|k-1}+K_{k}\left[y_{measured}-H{\bar{x}}_{k|k-1}\right]$$
  (e) Error covariance estimation:
    (10) $$P_{k|k}=\left[I-K_{k}H\right]P_{k|k-1}$$
  In order to increase the performance required to fulfill hard real-time constraints, the software components embedded in low-cost devices have been optimized in the following way: (a) use temporary variables to store complex calculations that are used multiple times along the code without changing the values; (b) reduce the number of calculations when handling matrices by expanding and analyzing the values prone to change; and (c) optimize the memory and instantiation time by passing function arguments as reference instead of value copies.
2. Second, an Unscented Kalman estimator was designed. Similarly to the previous estimator the inputs are the actual roll rate, time and lateral acceleration. The formulas implemented for the calculations are shown below [10]:
  (a) Calculate weights:
    (11) W0m=kk(n+k)(n+k)Wim=112(n+k)2(n+k);i=1,…,2nWic=kk(n+k))+(1-α2+β)(n+k))+(1-α2+β)Wic=112(n+k)2(n+k);i=1,…,2n
    where α is the distribution of the sampling points around the state mean, $\bar{x}$, β is used to incorporate prior knowledge of the distribution of $\bar{x}$, *n* is the dimension of $\bar{x}$, and *k* is a scaling parameter:
    $$k=\alpha^{2}\left(n+\epsilon \right)-n$$
    ϵ is usually set to 0.
  (b) Calculate sigma points:
    (12) $${\bar{x}}_{k|k}^{a}=\left[{\bar{x}}_{k|k}\;\;\;0\right]$$
    (13) $$P_{k|k}^{a}=\left[\begin{array}{ccc} P_{k|k} & 0 & 0\\ 0 & Q & 0\\ 0 & 0 & R \end{array}\right]$$
    (14) $$X_{k}^{a}=[{\bar{x}}_{k}^{a}\;\;\;{\bar{x}}_{k}^{a}+(\sqrt{\left(n+k\right)P_{xx}^{a}}\;\;\;{\bar{x}}_{k}^{a}-\left(\sqrt{\left(n+k\right)P_{xx}^{a}}\right]$$
  (c) Prediction of state:
    (15) $$X_{i,k+1|k}{=f(X}_{i,k|k}^{a})$$
    (16) $${\tilde{x}}_{k+1|k}^{}=\sum_{i=0}^{2n}W_{i}^{m}\cdot X_{i,k|k}^{}$$
  (d) Prediction of error covariance:
    (17) $$P_{k+1|k}=\sum_{i=0}^{2n}W_{i}^{c}\cdot \left(X_{i,k+1|k}^{}-{\tilde{x}}_{k+1|k}^{}\right)\cdot {\left(X_{i,k+1|k}^{}-{\tilde{x}}_{k+1|k}^{}\right)}^{T}$$
  (e) Prediction of observations:
    (18) $$Y_{i,k+1|k}{=h(X}_{i,k|k}^{a})$$
    (19) $${\tilde{y}}_{k+1|k}^{}=\sum_{i=0}^{2n}W_{i}^{m}\cdot Y_{i,k+1|k}$$
  (f) Innovation covariance:
    (20) $$P_{YY,k+1|k}=R+\sum_{i=0}^{2n}W_{i}^{c}\cdot \left(Y_{i,k+1|k}-{\tilde{y}}_{k+1|k}^{}\right)\cdot {\left(Y_{i,k+1|k}-{\tilde{y}}_{k+1|k}^{}\right)}^{T}$$
  (g) Cross correlation matrix:
    (21) $$P_{XY,k+1|k}=R+\sum_{i=0}^{2n}W_{i}^{c}\cdot \left(X_{i,k+1|k}^{}-{\tilde{x}}_{k+1|k}^{}\right)\cdot {\left(Y_{i,k+1|k}-{\tilde{y}}_{k+1|k}^{}\right)}^{T}$$
  (h) Kalman gain:
    (22) $$K_{k+1}=P_{XY,k+1|k}P_{YY,k+1|k}^{-1}$$
  (i) Error covariance estimation:
    (23) $$P_{k+1|k+1}=P_{k+1|k}-K_{k+1}P_{XY,k+1|k}K_{k+1}^{T}$$
  (j) State estimation:
    (24) $${\tilde{x}}_{k+1|k+1}^{}={\tilde{x}}_{k+1|k}^{}+K_{k+1}\left(y_{meas}-{\tilde{y}}_{k+1|k}^{}\right)$$
  In this case, the Cholesky transform was implemented in a separate component due to enhance the reuse of this calculation in further research works. This estimated includes the same optimizations as the previous case, being more relevant due to the increased complexity of the calculations stated for Unscented Kalman Filters.

In this case, the values of Q and R are:

(25) $$Q=\left[\begin{array}{cc} 5 & 0\\ 0 & 10 \end{array}\right]$$

(26) R=0.01

### 2.2. Experiments Specification

In order to evaluate the accuracy and performance of roll angle estimations provided by low-cost experimental kits, the following hypothesis were defined:

- H1: The roll angle estimation based on linear and unscented Kalman filters are similar than the actual roll angle values directly measured from the ground kit.
- H2: The performance of the roll angle estimation based on linear and unscented Kalman filters fulfills the constraints of hard real time processing providing, at least, results at a sampling rate of 50 Hz.

**Figure 6:**
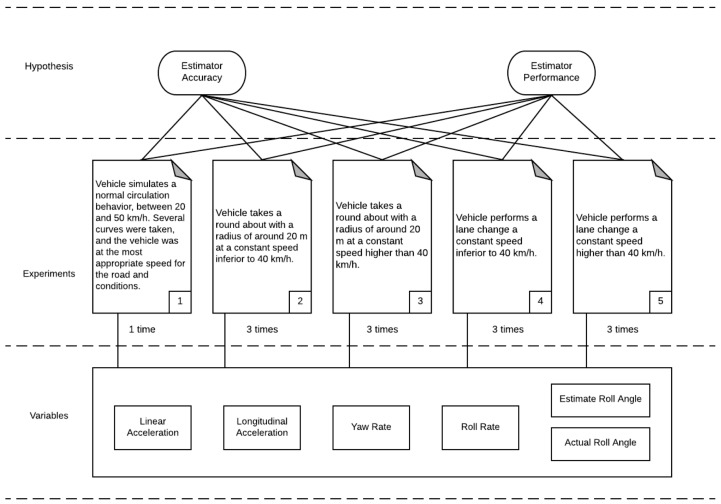
Defined experiments.

It was necessary to consider maneuvers such as regular driving situations, J-Turn and lane change to evaluate properly the previously stated hypothesis. Figure 6 summarizes the experiments defined.

### 2.3. Data Processing

As defined in the experimental testbed, when the Stop experiment signal is invoked, the Ecosystem Manager stores the information gathered by each kit in a CSV file having a name that includes the execution date and time and experimental kit identifier. The information included in each file depends on the considered kit.

The ground truth provides the GPS coordinates where the measure is obtained, the measure time stamp, roll and yaw rate, longitudinal and lateral acceleration and the actual roll angle obtained from the VBOX dual antenna.

The low-cost experimental kits provide exactly the same value but the roll angle value corresponds to the data generated by the estimators based on linear and unscented Kalman filters.

The actual roll angle obtained from the ground truth kit was compared with the values calculated by the roll angle estimation based on linear and unscented Kalman filters to determine the accuracy of the values obtained from the estimators. The results obtained during the experiments execution are presented in Section 3.

### 2.4. Threats to Validity

Several threats must be considered in order to determine the validity of the results obtained in this research work.

Regarding the experiments definition, it is necessary to consider all the issues preventing the experiments replication and the results generalization. The considered issues were related to:

1. The road conditions. The road considered in this research work has not slope or gradient. Nevertheless the maneuvers were repeated in different directions at the speed specified for each experiment.
2. The vehicle conditions. The threat in this category is related to the equipment conditions. Considering the recommendations provided in [14], the Racelogic IMU and the low-cost sensors were located in the vehicle’s center of mass.
3. The type of sensors and controllers considered. The threat in this category is related to the representativeness of the sensors embedded in the low-cost experimental kits. Nevertheless, it is important to remark that all the sensors considered are available on the market and they have an average price and quality. It can be foreseen that the sensors performance will improve in the coming years, so the results from the experiments execution can be better in the short term.

Regarding the experiments implementation, it is necessary to consider the relevant issues to prevent the errors introduction due to incorrect experiments execution. The considered issues in this area were:

1. The lack of precision in the measures obtained from the low-cost kits. As indicated in [14], the low-cost experimental kits provides data with the required precision. This precision is obtained when the corresponding calibrations are carried out in static conditions. Even more, to prevent errors from the specific sensors, two different units of each low-cost experimental kit type (Raspberry Pi and Intel Edison) were used.
2. The possible errors introduced by the sensor drivers and the middleware components considered in the IoT architecture designed to implement the experimental testbed. This threat was mitigated designing and implementing an automated unit testing plan to assure before the experiments execution, that these software components are free from critical bugs. The testing plan is automated executed before the deployment of the software components in the controller hardware in each low-cost experimental kit.
3. The possible errors in the execution of the maneuvers considered in each experiment. The mitigation of this threat consisted on the repetition of each experiment. Each maneuver was repeated, at least, three times.

## 3. Results

To perform the experimental validation corresponding to this research work, a Mercedes Sprinter van equipped with the low-cost devices and the Racelogic VBOX, as it is described in Section 2, was used. To evaluate both accuracy and processing time for the analyzed low-cost devices, two maneuvers were carried out, J-Turn and lane change. Also, a third test type was conducted in order to verify the devices behavior under normal circulation situations.

For the estimators presented in Section 2, the initial values for the state vector and the error covariance matrix are:

(27) $$x_{0}=\left[\begin{array}{c} 0\\ 0 \end{array}\right]$$

(28) $$P_{0}=\left[\begin{array}{cc} 1 & 0\\ 0 & 1 \end{array}\right]$$

### 3.1. J-Turn

**Figure 7:**
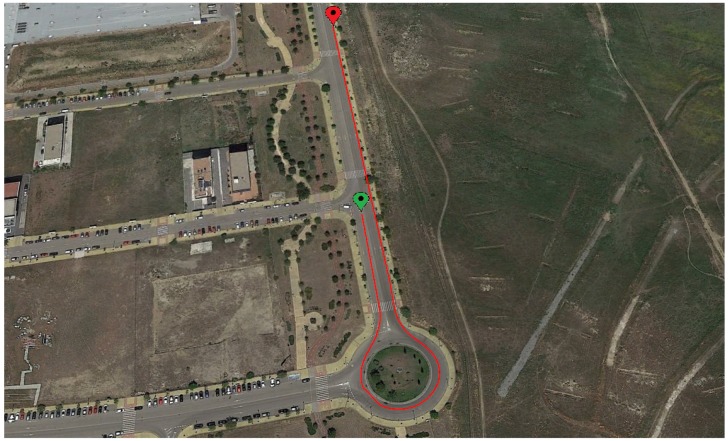
Test 1: J-turn trajectory (Map scale 1:2100 cm).

**Figure 8:**
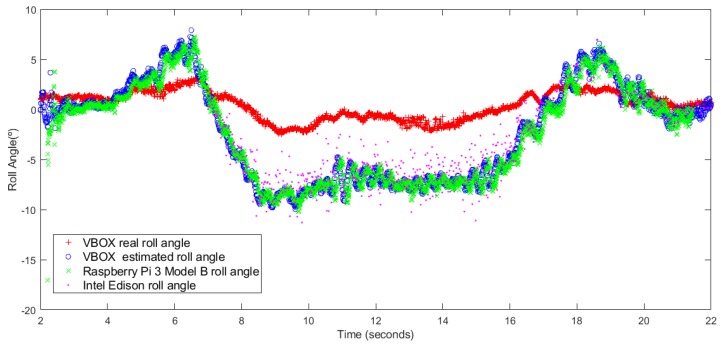
Test 1 (KF): Roll angle obtained from the dual antenna of VBOX (**red points**), estimated with Raspberry pi (**green points**), with Intel Edison (**pink points**) and with the IMU of VBOX (**blue points**).

**Figure 9:**
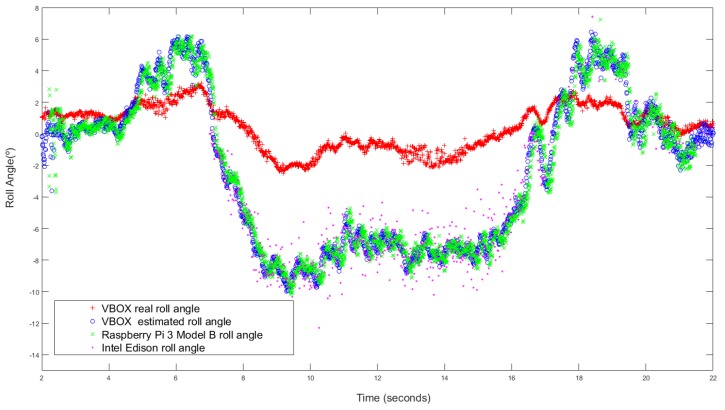
Test 1 (UKF): Roll angle obtained from the dual antenna of VBOX (**red points**), estimated with Raspberry pi (**green points**), with Intel Edison (**pink points**) and with the IMU of VBOX (**blue points**).

The first maneuver is a J-Turn, this maneuver is performed in a 22 m diameter roundabout at a speed close to 40 km/h (see Figure 7). Figure 8 and Figure 9 show the roll angle estimated by the Raspberry Pi 3 Model B (Green), Intel Edison (Pink) and with the information provided by the VBOX IMU (Blue). The roll angle measured with the VBOX GPS dual antenna (Red) has been used as ground truth in order to verify the accuracy of the devices and verify that the estimation is not affected by the low-cost components. In the three cases, estimations are very similar and the results show that the error comes principally from the estimator and not from the accuracy of the low-cost sensors. Also, in all the cases, the results show that there is a road bank in the path corresponding to the maneuver.

The root mean square (RMS), the norm and maximum errors have been calculated in order to quantify the accuracy of the estimation. The norm error has been calculated as follows [7]:

(29) $$E_{t}=\frac{\varepsilon_{t}}{\sigma_{t}}\times 100,$$

where

(30) $$\begin{array}{c} \varepsilon_{t}^{2}=\int_{0}^{T}{\left(\phi_{GT}-\phi_{lc}\right)}^{2}dt\\ \sigma_{t}^{2}=\int_{0}^{T}{\left(\phi_{GT}-\mu_{GT}\right)}^{2}dt \end{array}$$

$\phi_{GT}$ represents the ground truth data, $\phi_{lc}$ represents the low-cost sensor data and $\mu_{GT}$ is the mean value of the ground truth data obtained during the period T.

Table 3 collects the previous values. The results show that errors are very similar in both devices and the estimated roll angle using VBOX IMU data. The norm error from Intel Edison is higher than the Raspberry Pi and the VBOX IMU (about 7%) but for the maximum error the data from Raspberry Pi is higher than the other two values (about 12°). With UKF the errors are smaller in the three devices (about 7% for norm error, 0.2${}^{\circ }$ for RMS error and 0.3${}^{\circ }$ for maximum error).

These two estimators must work under hard real time constraints. For the given case, 20 ms is the maximum processing time permitted to transform the inputs and to apply the estimator. This sampling rate (50 Hz) is forced by the low-cost sensors.

Table 4 contains both processing time for Raspberry Pi 3 Model B and Intel Edison when computing KF and UKF. The mean and maximum processing time are calculated in order to quantify both devices performance. The devices stability has been determined with the processing time mean deviation. Results show that processing times for Intel Edison are higher than the Raspberry Pi 3 model B ones. Concerning to estimators, the mean processing time is higher in the case of UKF than KF (27$\times 10^{-6}$ s for Rasbperry Pi 3 Model B and 46$\times 10^{-6}$ s for Intel Edison). The difference between both devices are 3.5$\times 10^{-6}$ s with KF and 23$\times 10^{-6}$ s with UKF for the mean processing time, and 0.3$\times 10^{-3}$ s with KF and 0.6$\times 10^{-3}$ s with UKF for the maximum processing time. Concerning mean deviation, the difference is about 0.7$\times 10^{-6}$ s with KF and 9$\times 10^{-6}$ s with UKF.

### 3.2. Lane Change

**Figure 10:**
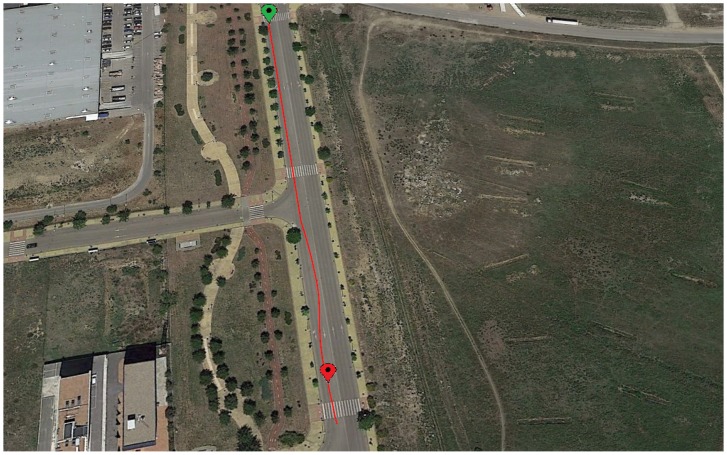
Test 2: Line change trajectory (Map scale 1:2100 cm).

**Figure 11:**
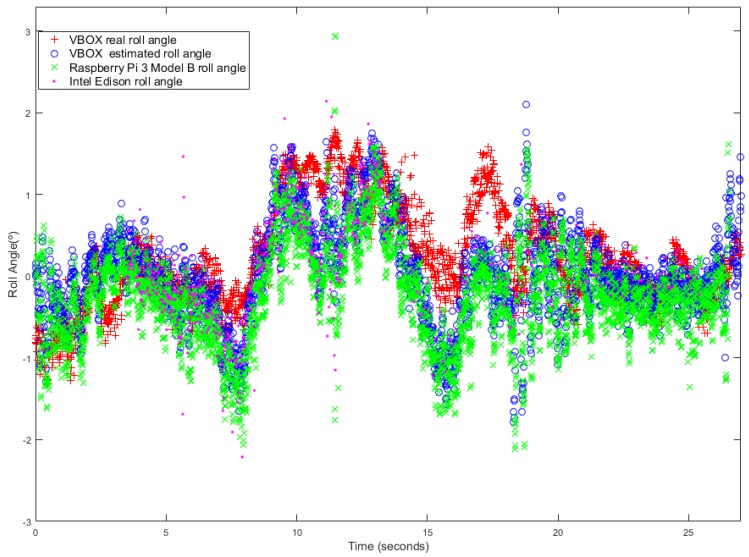
Test 2 (KF): Roll angle obtained from the dual antenna of VBOX (**red points**), estimated with Raspberry pi (**green points**), with Intel Edison (**pink points**) and with the IMU of VBOX (**blue points**).

**Figure 12:**
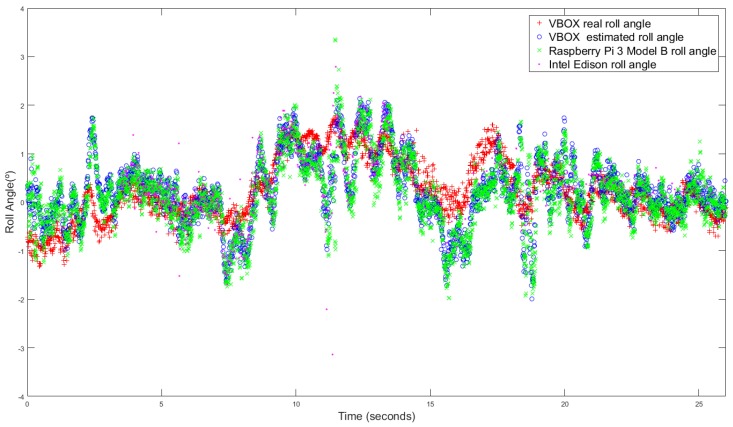
Test 2 (UKF): Roll angle obtained from the dual antenna of VBOX (**red points**), estimated with Raspberry pi (**green points**), with Intel Edison (**pink points**) and with the IMU of VBOX (**blue points**).

The second maneuver is a lane change, this was performed at a speed close to 50 km/h (see Figure 10). Figure 11 and Figure 12 show the roll angle estimated by the Raspberry Pi 3 Model B (green), Intel Edison (pink) and with the information provided by the VBOX IMU (blue) using a KF and in an UKF respectively. As in the previous maneuver, the roll angle measured with the VBOX GPS dual antenna (red) has been used as ground truth in order to verify the devices accuracy and to assess that the resulting estimation is not affected by the low-cost sensors and devices. In the three cases, the estimation is very similar and the results show that the error comes mainly from the estimator itself and not from the accuracy of the low-cost sensors. Another source of this error may be due to irregularities of the road as it happens during the time period between 15 s and 20 s.

The root mean square (RMS), the norm and maximum errors have been calculated in order to quantify the accuracy of the estimation. Table 5 shows the results. Errors are higher for Raspberry Pi 3 Model B than for Intel Edison. Concerning the norm and RMS error, the difference is about 15% and 0.15${}^{\circ }$ for Kalman Filter and 10% and 0.11${}^{\circ }$ for UKF. For maximum error the difference is about 0.1${}^{\circ }$ for KF and 2.2${}^{\circ }$ for UKF. The results for the estimated roll angle using VBOX IMU data are very similar, and in some cases, they are higher than Intel Edison ones.

Similarly to the previous maneuver, Table 6 shows both processing time for Raspberry Pi 3 Model B and Intel Edison when computing KF and UKF. The mean and maximum processing time are calculated in order to quantify the performance of both devices. The stability of the devices has been calculated throw the mean deviation of the processing time. Results show that processing times for Intel Edison are lower than the Raspberry Pi 3 model B ones. Concerning to estimators, the mean processing time is higher for UKF than for KF (30$\times 10^{-6}$ s for Raspberry Pi 3 Model B and 45$\times 10^{-6}$ s for Intel Edison). The difference between both devices are 1.25$\times 10^{-6}$ s with KF and 11$\times 10^{-6}$ s with UKF for the mean processing time and 14$\times 10^{-3}$ s with KF and 6.5$\times 10^{-3}$ s with UKF for the maximum processing time. Concerning mean deviation, the difference is about 10$\times 10^{-6}$ s with KF and 24$\times 10^{-6}$ s with UKF.

### 3.3. Standard Circulation

**Figure 13:**
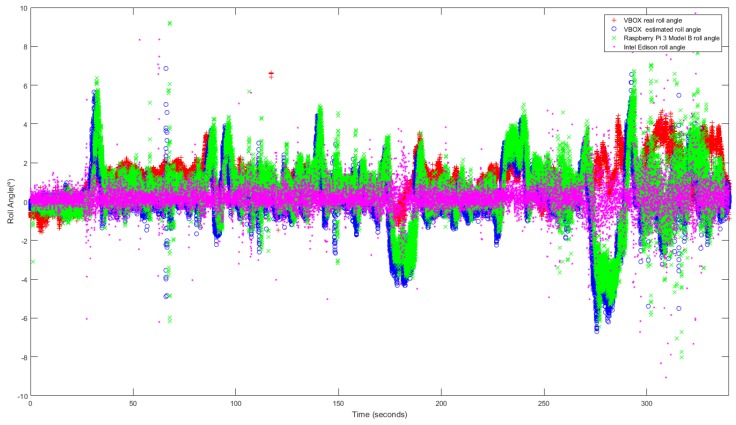
Test 3 (KF): Roll angle obtained from the dual antenna of VBOX (**red points**), estimated with Raspberry pi (**green points**), with Intel Edison (**pink points**) and with the IMU of VBOX (**blue points**).

**Figure 14:**
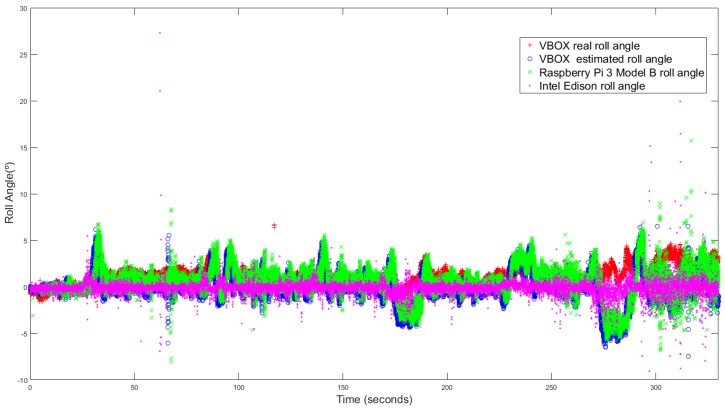
Test 3 (UKF): Roll angle obtained from the dual antenna of VBOX (**red points**), estimated with Raspberry pi (**green points**), with Intel Edison (**pink points**) and with the IMU of VBOX (**blue points**).

The last test is performed taking low and medium velocity with typical maneuvers and smooth movements. This test is carried out under usual circulation conditions. Figure 13 and Figure 14 show the roll angle estimated by the Raspberry Pi 3 Model B (green), Intel Edison (pink) and with the information provided by the VBOX IMU (blue), by using a KF and an UKF respectively. The roll angle measured with the VBOX GPS dual antenna (Red) has been used as ground truth in order to verify the accuracy of the devices and to verify that the resulting estimation is not affected by the low-cost sensors nor devices. In all the three cases, the error is very similar and higher than in previous tests. These devices are very sensitive to the noise [14] and this test type is prone to noise.

The root mean square (RMS), the norm and maximum errors have been calculated in order to quantify the accuracy of the estimation. In Table 7 the results are given, the errors are very similar except for the maximum error, where the Intel Edison has a 55.81${}^{\circ }$ of error for KF and 43,68 for UKF, this error is due to the noise that causes atypical data Concerning the norm and RMS error, the difference is about 0.06% and 0.12${}^{\circ }$ for Kalman Filter and 4% and 0.15${}^{\circ }$ better for UKF for Intel Edison and for Raspberry Pi the difference is about 5% and 0.17${}^{\circ }$ for Kalman Filter and 7% and 0.18${}^{\circ }$ better for UKF. The results of the estimated roll angle using VBOX IMU data are very similar, and in some test are higher than the both Raspberry Pi and Intel Edison.

Similarly to the previous maneuver, Table 8 shows both processing time for Raspberry Pi 3 Model B and Intel Edison when computing KF and UKF. The mean and maximum processing time are calculated in order to quantify the performance of both devices. The stability of the devices has been calculated throw the mean deviation of the processing time. Results show that processing times for Intel Edison are lower than the Raspberry Pi 3 model B ones. Concerning to estimators, the mean processing time is higher for UKF than for KF (26$\times 10^{-6}$ s for Rasbperry Pi 3 Model B and 47$\times 10^{-6}$ s for Intel Edison). The difference between both devices are 3.2$\times 10^{-6}$ s with KF and 24$\times 10^{-6}$ s with UKF for the mean processing time and 6$\times 10^{-3}$ s with KF and 7.7$\times 10^{-3}$ s with UKF for the maximum processing time. Concerning mean deviation, the difference is about 1$\times 10^{-6}$ s with KF and 9$\times 10^{-6}$ s with UKF.

## 4. Discussion

The results discussion is focused on the performance of the low-cost devices and the accuracy of the estimations obtained.

### 4.1. Accuracy

As expected, the roll angle estimations provided by the Unscented Kalman are better than the ones obtained using Linear Kalman. The RMS error for all the tests obtained by both estimators in comparison with the ground truth values are between 0.1 and 2.2 degrees. So, it can be concluded that the estimations are accurate enough for the expected use in RSC.

Nevertheless, it is necessary to remark that the results calculated by these estimators are less precise than the provided by Neural Network based estimators. However, Kalman based estimators do not need a previous training and are tight to an specific model allowing its direct usage in a variety of vehicles.

Also, it can be indicated that Kalman Filters based estimators are less sensitive to noise than Neural Networks based ones.

Finally, as future work, it is planned to create more complex estimators based on a combination of Neural Networks and Kalman Filters because the performance of software components implementing these techniques in low-cost devices seems to be high enough to support both of them respecting hard real time constraints.

### 4.2. Processing Capability

The temporal performance and real time constraints are main aspects to consider in order to integrate estimators and controllers in embedded low-cost devices. The results show that the processing time to get the data, execute its normalization, perform the roll angle estimation via ANN and the denormalization of the outcome, is four orders of magnitude lower than the required sample rate threshold of 20 ms.

The average processing times are 5.16$\times 10^{-6}$ s (KF) and 51.83$\times 10^{-6}$ s (UKF) for Intel Edison and 1.82$\times 10^{-6}$ s (KF) and 28.64$\times 10^{-6}$ s (UKF) for Raspberry Pi 3 Model B.

This performance will permit the integration of Neural Networks and Kalman filters to reduce the noise present in data gathered from low cost sensors.

Several optimizations were carried out to enhance this performance, essentially:

- Usage of standard libraries from C++, which allow a straightforward compilation in almost any development board.
- Reduction of the number of operations when handling matrices by expanding and analyzing the specific resulting values prone to change (algorithmic optimization).
- Optimization of memory usage and instantiation time by passing function arguments as reference instead of value copies, and by multiple revisions of source code to keep it clean and simple.

## 5. Conclusions

As results presented in Section 3 indicate, low-cost experimental kits based on IoT architectures and including estimators based on Kalman filters provide accurate roll angle estimation. Moreover, an effective design and development of software components applying these techniques is essential to fulfill hard real time restrictions when embedding this components in low cost devices such as Raspberry Pi 3 Model B and Intel Edison to estimate roll angle. Scalable, versatile, maintainable and efficient IoT architectures for commercial vehicles can be implemented using the software design and results obtained in this research work. As stated before, the performance levels demonstrated in this research work envisage that more complex estimators based on the combination of Kalman filters, Neural Networks and other techniques such as deep learning algorithms can provide better results.

## Figures and Tables

**Table 1:** Variables glossary.

Variable	Definition	Variable	Definition
$x_{k}$	State vector	$I_{xx}$	Sprung mass moment of inertia
$\phi_{k}$	Vehicle roll angle	$m_{s}$	Sprung mass
${\dot{\phi }}_{k}$	Vehicle roll rate	$h_{cr}$	Sprung mass height about the roll axis
$a_{ym,k}$	Vehicle lateral acceleration	$C_{r}$	Total torsional damping
$y_{k}$	Observation vector	$K_{r}$	Stiffness coefficient
*k*	Instant time	$T_{s}$	Sample time
*g*	Acceleration due to gravity	$P_{k}$	Error covariance matrix
*R*	Covariance matrix of the measurement noise	*Q*	Process noise covariance matrix
*K*	Kalman gain matrix	*H*	Observation matrix

**Table 2:** Vehicle model variables.

Variable	Definition	Value
$I_{xx}$	Sprung mass moment of inertia	751.16 kg m${}^{2}$
$m_{s}$	Sprung mass	1700 kg
$h_{cr}$	Sprung mass height about the roll axis	0.25 m
$C_{r}$	Total torsional damping	3538.08 N m/rad
$K_{r}$	Stiffness coefficient	18,438.02 N/m${}^{2}$

**Table 3:** Test 1: Errors of estimated roll angle using KF and UKF on Raspberry Pi and Intel Edison compared with the IMU from VBOX (ground truth).

	Roll Angle					
	Norm Error (%)		RMS Error (${}^{\circ }$)		Maximum Error (${}^{\circ }$)	
	KF	UKF	KF	UKF	KF	UKF
Raspberry Pi 3 Model B	43.38	42.29	2.41 ± 0.35	2.35 ± 0.04	19.32	19.06
Intel Edison	54.97	51.18	2.54 ± 0.21	5.37 ± 0.07	8.94	6.87
Racelogic VBOX IMU	48.89	45.51	2.33 ± 0.21	2.17 ± 0.32	6.38	6.66

**Table 4:** Test 1: Processing time using KF and UKF on Raspberry Pi and Intel Edison.

	Processing Time					
	Maximum (s)		Mean (s)		Mean Deviation (s)	
	KF	UKF	KF	UKF	KF	UKF
Raspberry Pi 3 Model B	0.29$\times 10^{-3}$	0.77$\times 10^{-3}$	1.82$\times 10^{-6}$	28.64$\times 10^{-6}$	0.96$\times 10^{-6}$	11.25$\times 10^{-6}$
Intel Edison	6$\times 10^{-6}$	100$\times 10^{-6}$	5.16$\times 10^{-6}$	51.83$\times 10^{-6}$	0.27$\times 10^{-6}$	2.55$\times 10^{-6}$

**Table 5:** Test 2: Errors of estimated roll angle using KF and UKF on Raspberry Pi and Intel Edison compared with the IMU from VBOX (ground truth).

	Roll Angle					
	Norm Error (%)		RMS Error (${}^{\circ }$)		Maximum Error (${}^{\circ }$)	
	KF	UKF	KF	UKF	KF	UKF
Raspberry Pi 3 Model B	120.1	100.2	0.76 ± 0.035	0.63 ± 0.058	3.21	2.76
Intel Edison	105.4	89.9	0.61 ± 0.051	0.52 ± 0.078	3.11	4.78
Racelogic VBOX IMU	106.1	104.23	0.66 ± 0.012	0.65 ± 0.05	2.3	3.38

**Table 6:** Test 2: Processing time using KF and UKF on Raspberry Pi and Intel Edison.

	Processing Time					
	Maximum (s)		Mean (s)		Mean Deviation (s)	
	KF	UKF	KF	UKF	KF	UKF
Raspberry Pi 3 Model B	14.21$\times 10^{-3}$	6.76$\times 10^{-3}$	6.93$\times 10^{-6}$	374.77$\times 10^{-6}$	10.54$\times 10^{-6}$	26.93$\times 10^{-6}$
Intel Edison	88$\times 10^{-6}$	120$\times 10^{-6}$	5.25$\times 10^{-6}$	51.93$\times 10^{-6}$	0.41$\times 10^{-6}$	2.65$\times 10^{-6}$

**Table 7:** Test 3: Errors of estimated roll angle using KF and UKF on Raspberry Pi and Intel Edison compared with the IMU from VBOX (ground truth).

	Roll Angle					
	Norm Error (%)		RMS Error (${}^{\circ }$)		Maximum Error (${}^{\circ }$)	
	KF	UKF	KF	UKF	KF	UKF
Raspberry Pi 3 Model B	193.86	186.67	1.86	1.79	15.67	14.33
Intel Edison	198.97	189.46	1.91	1.82	55.81	43.68
Racelogic VBOX IMU	198.91	193.34	2.03	1.97	9.9	8.91

**Table 8:** Test 3: Processing time using KF and UKF on Raspberry Pi and Intel Edison.

	Processing Time					
	Maximum (s)		Mean (s)		Mean Deviation (s)	
	KF	UKF	KF	UKF	KF	UKF
Raspberry Pi 3 Model B	6.41$\times 10^{-3}$	8.436$\times 10^{-3}$	2.05$\times 10^{-6}$	28.49$\times 10^{-6}$	1.33$\times 10^{-6}$	12.27$\times 10^{-6}$
Intel Edison	0.054$\times 10^{-6}$	0.71$\times 10^{-6}$	5.21$\times 10^{-6}$	52.10$\times 10^{-6}$	0.35$\times 10^{-6}$	3.11$\times 10^{-6}$

## Abbreviations

The following abbreviations are used in this manuscript:

GPS	Global Positioning System
IMU	Inertial Measurement Unit
IoT	Internet Of Things
RMS	Root Mean Square
